# Core–shell Au@Ag nanoparticles as a high-performance SERS substrate for detection of multiple fungicides on mango surfaces

**DOI:** 10.1039/d6ra05546j

**Published:** 2026-09-17

**Authors:** Ziwen Zhang, Weiye Yang, Dongan Wu, Ping Liang, Hongyan Peng, Lijian Meng, Shihua Zhao

**Affiliations:** a College of Physics and Electronic Engineering, Hainan Normal University HaiKou 571158 China zsh@hainnu.edu.cn haha_weiye@163.com; b State Key Laboratory of Tropic Ocean Engineering Materials and Materials Evaluation 570228 Haikou China; c The Innovation Platform for Academicians of Hainan Province Haikou Hainan Province 571158 China; d CIETI/ISEP, Polytechnic of Porto Rua Dr António Bernardino de Almeida Porto 4249-015 Portugal

## Abstract

Fungicide residues pose significant risks to food safety and public health. Conventional detection methods are often time-consuming, expensive, and require complex sample preparation, limiting their applicability for rapid on-site detection. In this study, gold-core silver-shell nanoparticles (Au@Ag NPs) were synthesized using a seed-mediated growth method and employed as a high-performance surface-enhanced Raman scattering (SERS) substrate. The prepared substrate demonstrated a limit of detection (LOD) as low as 8.414 × 10^−14^ M for Rhodamine 6G (R6G) and an enhancement factor (EF) of 2.78 × 10^8^, exhibiting strong SERS enhancement performance, good reproducibility, and acceptable storage stability. The substrate was successfully applied to the rapid detection of three commonly used fungicides (thiram, thiabendazole, and difenoconazole) in standard solutions, achieving detection limits of 0.0047 ppm, 0.0011 ppm, and 0.105 ppm, respectively. It was also applied to detection on mango surfaces. The method was further evaluated using individually spiked mango surfaces, yielding recoveries of 92.24–108.43% for thiram, 92.80–107.64% for thiabendazole, and 82.47–94.85% for difenoconazole, with RSD values below 8%. Furthermore, simultaneous qualitative fingerprint identification of thiram, thiabendazole, and difenoconazole in a ternary mixture was demonstrated on spiked mango surfaces. The proposed SERS sensing strategy provides a rapid, sensitive, and reliable analytical method, presenting promising potential for the on-site screening of multiple pesticide residues in agricultural products.

## Abstract 2

Fungicide residues pose significant risks to food safety and public health.

## Graphical abstract

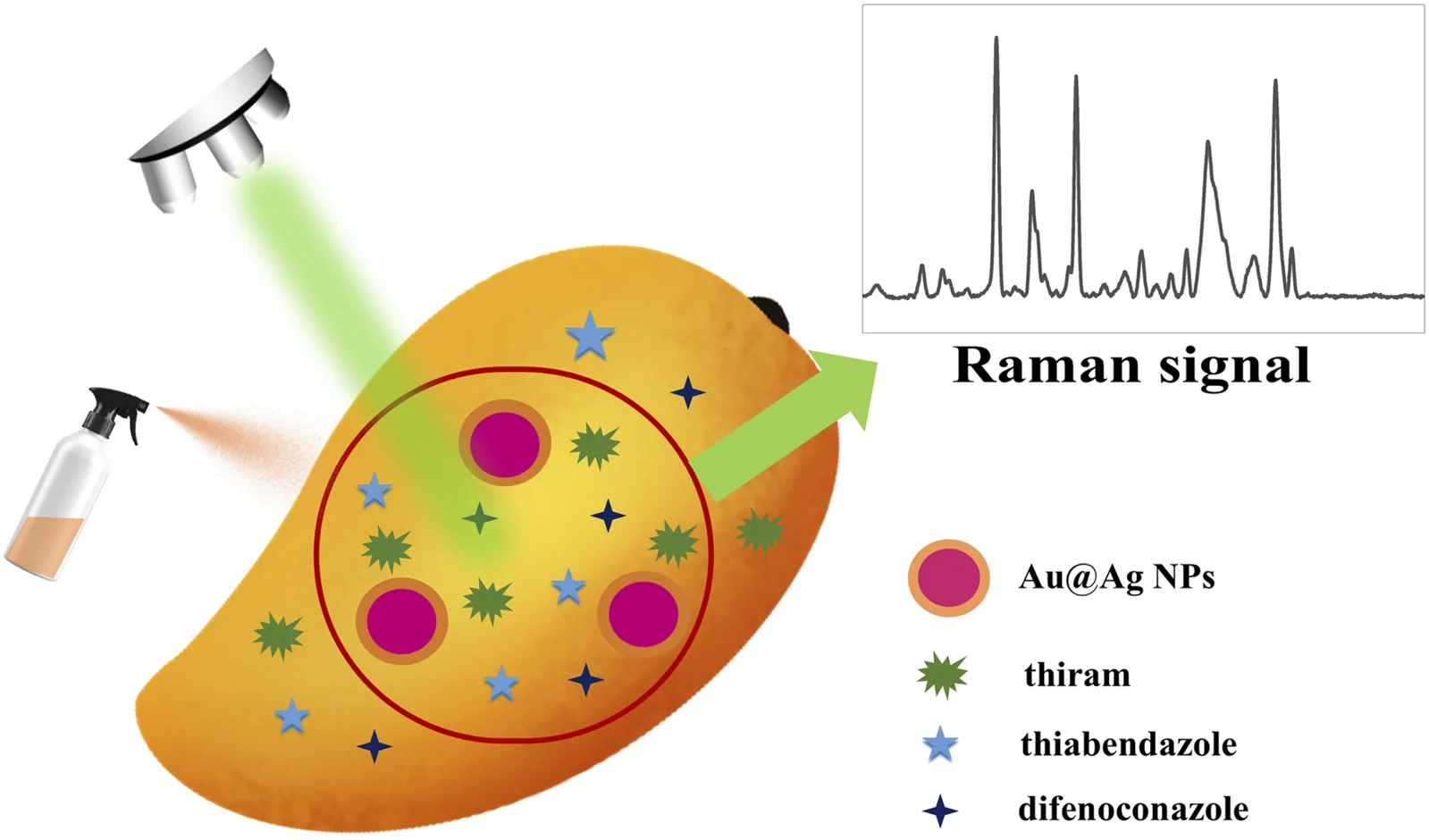

## 1 Introduction

In contemporary agricultural production systems, pesticides are essential for ensuring crop yield, disease prevention, and product quality.^1^ Fungicides, constituting over one-third of the global pesticide market, are extensively employed to control significant fungal diseases such as gray mold and powdery mildew in fruits and vegetables.^2^ However, excessive application during cultivation, post-harvest storage, and transportation inevitably leads to residue accumulation in agricultural products, thereby posing substantial risks to public health and ecosystems.^3,4^ Some fungicides have been confirmed as mutagenic, cytotoxic, or carcinogenic; therefore, fungicide residues have become a globally recognized critical issue in food safety.^5,6^

With its unique tropical climate, Hainan serves as a vital agricultural production region in China. The high humidity and temperature predispose crops to various fungal diseases, resulting in extensive fungicide application in local cultivation and post-harvest processes. As a major tropical agricultural base, Hainan faces dual challenges from intensive multi-cropping practices and complex post-harvest logistics, resulting in detectable excessive fungicide residues in certain products. This emerging food safety issue hampers sustainable agricultural development and poses ecological and public health risks in the region. Therefore, rigorous monitoring and comprehensive study of fungicide application and residue dynamics are critically important for guiding safe production and quality control practices.^7^

Among numerous fungicides, thiram, difenoconazole, and thiabendazole are three widely used agents associated with significant residue risks. Thiram, a dithiocarbamate primarily utilized for seed treatment and post-harvest preservation,^8,9^ can cause respiratory and kidney toxicity.^10^ Difenoconazole, a broad-spectrum systemic fungicide, has been linked to teratogenic effects.^11^ Thiabendazole, another common post-harvest fungicide, is classified by the United States Environmental Protection Agency (USEPA) as a potential carcinogen at high doses and is capable of disrupting endocrine function.^12–14^ Consequently, both the European Union and China have established strict maximum residue limits (MRLs) for these fungicides in foods.^15^

Currently, detection of fungicide residues, including thiram, difenoconazole, and thiabendazole, primarily relies on established analytical techniques. High-performance liquid chromatography (HPLC),^16^ gas chromatography-mass spectrometry (GC-MS),^17^ and liquid chromatography-mass spectrometry (LC-MS)^18^ are considered benchmark methods for fungicide residue analysis due to their exceptional sensitivity and accuracy. Additionally, enzyme-linked immunosorbent assay (ELISA) is widely employed for preliminary screening owing to its high throughput and specificity.^19^ However, these analytical techniques are unsuitable for rapid on-site detection due to their reliance on sophisticated instrumentation, lengthy sample preparation, substantial solvent consumption, and the need for skilled operators.^20–22^ Such limitations restrict their practical applicability in field settings like farms or markets. Consequently, there is an urgent need to develop novel analytical methods that are sensitive, simple, rapid, cost-effective, and suitable for field deployment to ensure effective food safety monitoring.

Over the past five years, SERS research on pesticide detection has increasingly emphasized addressing practical application challenges, including overcoming matrix effects, improving substrate stability, and enabling field deployment, thereby moving beyond the exclusive pursuit of ultra-high sensitivity.^23^ For instance, studies have developed renewable Ag/TiO_2_ composite substrates to enhance practicality,^24^ utilized low-cost biological templates (cicada wings) to fabricate AgNP-based substrates for detection,^25^ and reported SERS-based methods for rapid determination of multiple pesticide residues on mango surfaces.^26^ In addition, Au@Ag core–shell nanoparticles with tunable Ag-shell thickness have been systematically investigated for SERS enhancement and pesticide sensing, demonstrating the broad applicability of Au@Ag-based substrates in pesticide analysis.

Surface-enhanced Raman scattering (SERS) technology significantly amplifies the Raman signals of surface-adsorbed analytes *via* the localized surface plasmon resonance effect, achieving exceptional sensitivity down to the single-molecule level.^27,28^ Its combination of high sensitivity, fingerprint identification capability, rapid response, minimal sample preparation, and non-destructive analysis makes it particularly promising for food safety monitoring.^29,30^ The signal enhancement arises from electromagnetic and chemical mechanisms, with the electromagnetic mechanism, driven by localized “hot spots” at nanoscale gaps or tips, being critical for ultra-high sensitivity.^31^ Nonetheless, SERS performance critically depends on the plasmonic substrate. An ideal substrate should exhibit high enhancement factors (EF), excellent uniformity and reproducibility, stability, and straightforward preparation.^32,33^ Commonly used noble metals present a performance trade-off: silver nanoparticles (Ag NPs) offer strong SERS activity but suffer from oxidation and aggregation, resulting in poor stability; gold nanoparticles (Au NPs) are chemically stable but provide weaker enhancement.^34^ Thus, designing a substrate that simultaneously delivers high sensitivity, stability, and reproducibility remains a central challenge in translating SERS into practical applications.

Accordingly, this study develops an application-oriented SERS analytical workflow based on optimized Au@Ag NPs for fungicide analysis on mango surfaces. The colloidal substrate is applied directly to the fruit surface, enabling short-time residue extraction and analyte–nanoparticle interaction within the droplet, followed by direct Raman acquisition with minimal sample handling. The analytical performance of the method was evaluated through concentration-dependent measurements and quantitative calibration for thiram, thiabendazole, and difenoconazole individually, together with recovery analysis on individually spiked mango surfaces. In addition, a ternary-mixture experiment was performed to evaluate whether the characteristic Raman fingerprints of the three fungicides could be simultaneously distinguished on mango surfaces. From a practical perspective, this workflow reduces dependence on complicated extraction and matrix-cleanup procedures, shortens the overall analytical process, and enables direct analysis on the fruit surface. The combination of individual quantitative analysis and qualitative multicomponent fingerprint identification provides flexibility for both targeted determination and rapid multi-residue screening. This simplified surface-analysis strategy therefore provides a practical basis for rapid fungicide-residue screening in fruits and other agricultural products and may support the further development of portable SERS-based detection approaches.

## 2 Materials and methods

### 2.1 Materials and instruments

Chloroauric acid trihydrate, silver nitrate, trisodium citrate, and ascorbic acid were supplied by Shanghai Macklin Biochemical Technology Co., Ltd Rhodamine 6G (R6G) and thiabendazole were obtained from Shanghai Aladdin Biochemical Technology Co., Ltd. Methanol and absolute ethanol were used as received without further purification. All experiments were performed using ultrapure water. Mango samples used in this study were purchased from a local supermarket in Haikou City, Hainan Province, China.

### 2.2 Instruments

Ultraviolet-visible (UV-vis) absorption spectra of Au@Ag NPs were recorded using a dual-beam UV-vis spectrophotometer (Hitachi U-3900, Hitachi Ltd, Japan). The crystalline structure of Au@Ag nanoparticles was characterized by X-ray diffraction (XRD, Ultima-IV, Rigaku Corporation, Japan). Morphology and microstructure analyses were conducted using a transmission electron microscope (TEM, FEI Talos F200X) operating at 200 kV. All SERS spectra in this study were collected using a HORIBA Raman spectrometer (LabRAM Odyssey) equipped with a 532 nm excitation laser, a 50× objective lens, and a 600 gr mm^−1^ diffraction grating. Raman spectra were acquired using a 532 nm excitation laser with an output power of 100 mW, a 50× objective lens, an integration time of 5 s per spectrum, and three accumulations per sample.

### 2.3 Synthesis of Au@Ag NPs

The Au@Ag core–shell nanoparticles were synthesized through a two-step method. First, Au nanoparticle seeds were prepared using a classical citrate reduction procedure. Specifically, 60 mL of ultrapure water was heated to boiling in an Erlenmeyer flask under vigorous stirring at 1000 rpm. Then, 950 µL of aqueous chloroauric acid solution (5 g L^−1^) was rapidly introduced, and boiling was maintained for 2 min. Subsequently, 450 µL of trisodium citrate solution (1 wt%) was quickly added, and the mixture was continuously heated for another 6 min until the solution turned from pale yellow to wine red, indicating successful formation of Au nanoparticles. The resulting seed solution was cooled to room temperature and stored at 4 °C. To obtain Au seeds of different sizes, the amount of trisodium citrate solution was adjusted from 300 to 900 µL.

**Fig. 1:**
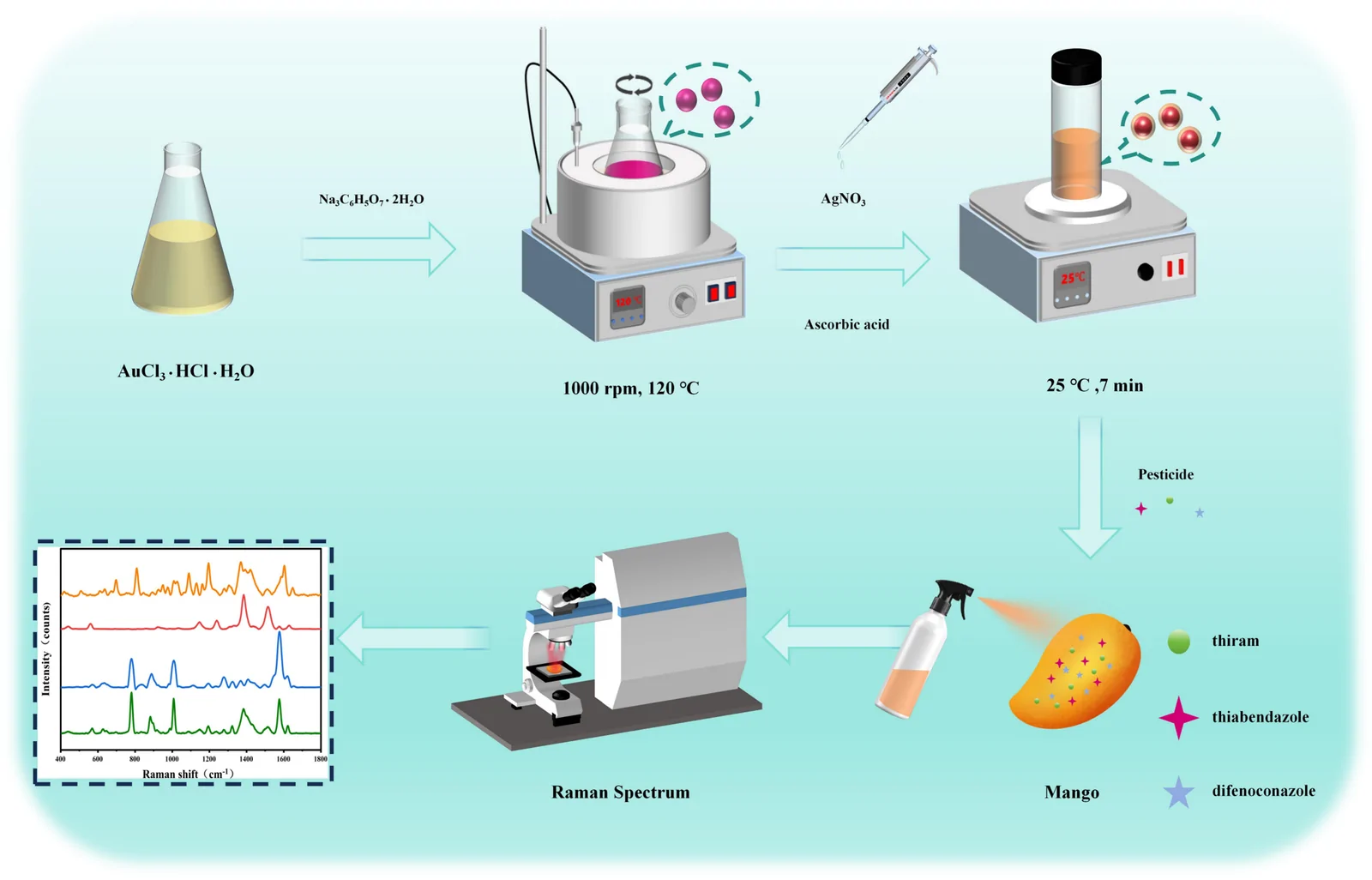
Schematic illustration of Au@Ag NPs preparation and the fungicides detection on mango surfaces.

Following seed synthesis, a silver shell was deposited onto the Au NPs. Typically, 3 mL of the Au NP seed solution was transferred into a vial, and 180 µL of ascorbic acid solution (10 mM) was added as a reducing agent under vigorous stirring. Subsequently, 180 µL of silver nitrate solution was added dropwise at intervals of approximately 30 s, followed by continuous stirring for 7 min to complete shell formation. To systematically investigate the influence of Ag-shell growth conditions on SERS performance, the volumes of ascorbic acid and silver nitrate solutions were simultaneously varied from 60 to 270 µL, resulting in Au@Ag nanoparticle formulations prepared under different Ag-shell growth conditions. Optimal nanoparticles were selected as substrates for subsequent experiments. The overall preparation process is illustrated in Fig. 1, where Au NPs of varying sizes were initially synthesized *via* the citrate reduction method by adjusting the amount of trisodium citrate, followed by controlled adjustment of the silver shell thickness through varied additions of silver nitrate and equal volumes of ascorbic acid.

### 2.4 Preparation of probe molecules

To evaluate the SERS performance, R6G was used as a Raman probe. R6G solutions with concentrations ranging from 10^−8^ M to 10^−13^ M were prepared and mixed with Au@Ag NP colloids in a 1 : 1 volume ratio. The mixtures were incubated in the dark at room temperature for 30 min.

To systematically evaluate the detection capability for multiple fungicides, thiabendazole, thiram, and difenoconazole were selected as target analytes. Stock solutions (100 ppm) of each fungicide were prepared in methanol and serially diluted with ultrapure water or methanol to prepare working solutions at concentrations ranging from 1 to 0.001 ppm for thiram, 5 to 0.005 ppm for thiabendazole, and 50 to 0.1 ppm for difenoconazole. Equal volumes of each fungicide solution were mixed with Au@Ag NP colloids, vortexed, and briefly equilibrated to achieve adsorption equilibrium before Raman analysis.

For experiments involving fungicide mixtures, single-fungicide standard solutions of thiabendazole (5 ppm), thiram (1 ppm), and difenoconazole (10 ppm) were combined in equal volume ratios to create a composite solution. Fresh mangoes of similar size and maturity without any prior treatment were sprayed with the mixed solution on approximately 1 cm^2^ areas of the peel, followed by air-drying at room temperature. For detection, the Au@Ag NP colloid was applied using a micropipette to fully cover the targeted area and allowed to interact for 2 min, enabling the spreading droplet to extract fungicide residues from the dried spot into the probed volume, thus minimizing potential sample inhomogeneities due to drying. Raman spectra were then directly acquired from the droplet-covered regions.

### 2.5 Recovery analysis

Recovery experiments were performed separately for thiram, thiabendazole, and difenoconazole using individually spiked mango surfaces. Three spiking levels were evaluated for each fungicide: 0.1, 0.5, and 1.0 ppm for thiram and thiabendazole, and 1, 5, and 10 ppm for difenoconazole. Three replicate measurements were performed at each concentration level using the same SERS measurement procedure described above. The detected concentrations were calculated from the corresponding calibration equations established using individually spiked mango surfaces, based on the characteristic SERS intensities at 1384 cm^−1^ for thiram, 1577 cm^−1^ for thiabendazole, and 808 cm^−1^ for difenoconazole. Recovery was calculated as the ratio of the detected concentration to the corresponding spiked concentration, and precision was expressed as the relative standard deviation (RSD) of the triplicate measurements.

## 3 Results and discussion

### 3.1 UV-vis absorption spectra analysis

**Fig. 2:**
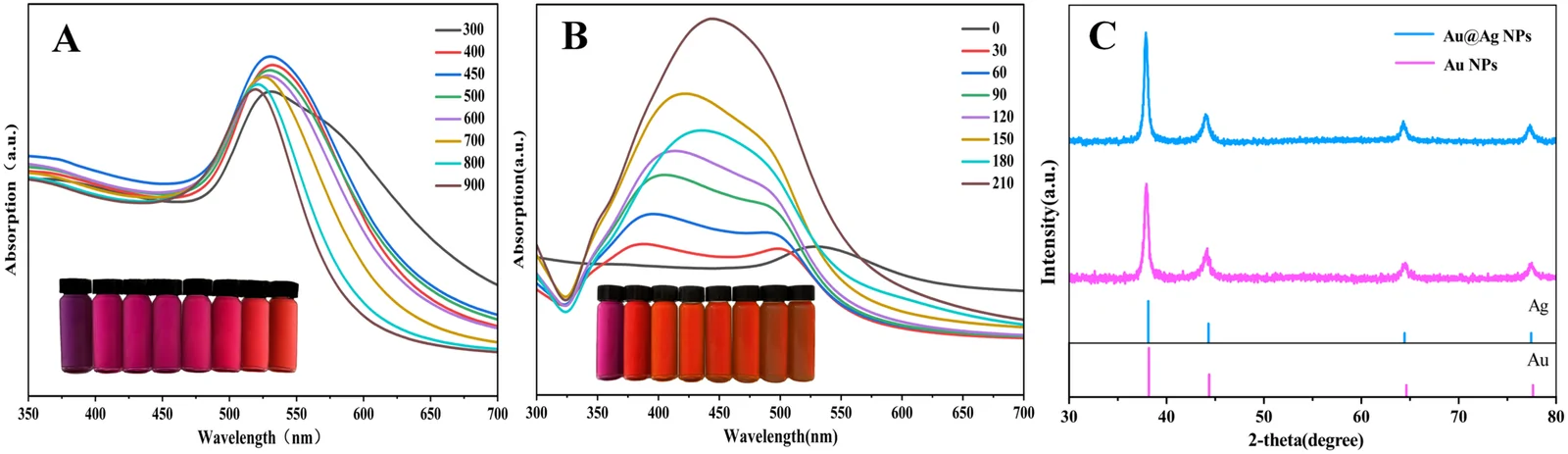
(A) UV-vis absorption spectra of Au NPs; (B) UV-vis absorption spectra of Au@Ag NPs; (C) XRD patterns of Au NPs and Au@Ag NPs.

Fig. 2(A) presents the UV-vis absorption spectra of Au NPs with different particle sizes. As the amount of trisodium citrate increased, the color of the synthesized Au NPs transitioned from dark purple to light red, indicating a gradual reduction in particle size. Correspondingly, the UV-vis absorption peak exhibited a blue shift as nanoparticle size decreased.^35^ In this study, Au NPs prepared with 300 µL trisodium citrate exhibited an absorption maximum at 532 nm. When trisodium citrate was increased to 900 µL, the peak shifted 13 nm toward shorter wavelengths. Among the eight Au NP sizes obtained, the sample synthesized with 450 µL trisodium citrate displayed the strongest absorption intensity, peaking around 530 nm. Therefore, these Au NPs were selected as seed materials for the subsequent preparation of Au@Ag NPs.

To systematically investigate the effect of Ag shell thickness on the UV-vis absorption properties of Au@Ag NPs, core–shell nanoparticles with varying shell thicknesses were synthesized (Fig. 2(B)). Increasing the silver nitrate and ascorbic acid amounts resulted in a gradual thickening of the silver shell.^36^ The corresponding UV-vis absorption spectra are presented in Fig. 2(B). The addition of 30 µL silver nitrate resulted in two distinct absorption peaks at 380 nm and 500 nm, corresponding respectively to the surface plasmon resonances of the Ag shell and the Au core.^37^

### 3.2 XRD analysis

To further confirm the successful synthesis of Au@Ag NPs, X-ray diffraction (XRD) analysis was performed on both Au NPs and Au@Ag NPs, as shown in Fig. 2(C). The XRD pattern of Au NPs exhibited four distinct diffraction peaks at 37.9°, 44.1°, 64.3°, and 77.3°, corresponding to the (111), (200), (220), and (311) crystal planes of face-centered cubic (fcc) gold, respectively.^38^ Among these, the (111) reflection displayed the sharpest and most intense peak, indicating high crystallinity of the Au seeds. Due to the very similar lattice constants of gold and silver, the diffraction peaks of Au@Ag NPs cannot be directly distinguished from those of pure Au or Ag. For reference, standard diffraction patterns of Ag and Au are provided at the bottom of Fig. 2(C). The Ag peaks are slightly shifted toward lower angles (a “blue shift”) relative to the Au peaks. A corresponding low-angle shift is also observable in the XRD patterns of Au@Ag NPs compared with pure Au NPs, confirming successful deposition of the Ag shell. Moreover, diffraction peaks of Au@Ag NPs are notably sharper, demonstrating that the epitaxially grown Ag shell on the gold core possesses high crystallographic quality, thus supporting the formation of the core–shell structure.

### 3.3 Crystallographic and elemental characterization of Au NPs and Au@Ag NPs

**Fig. 3:**
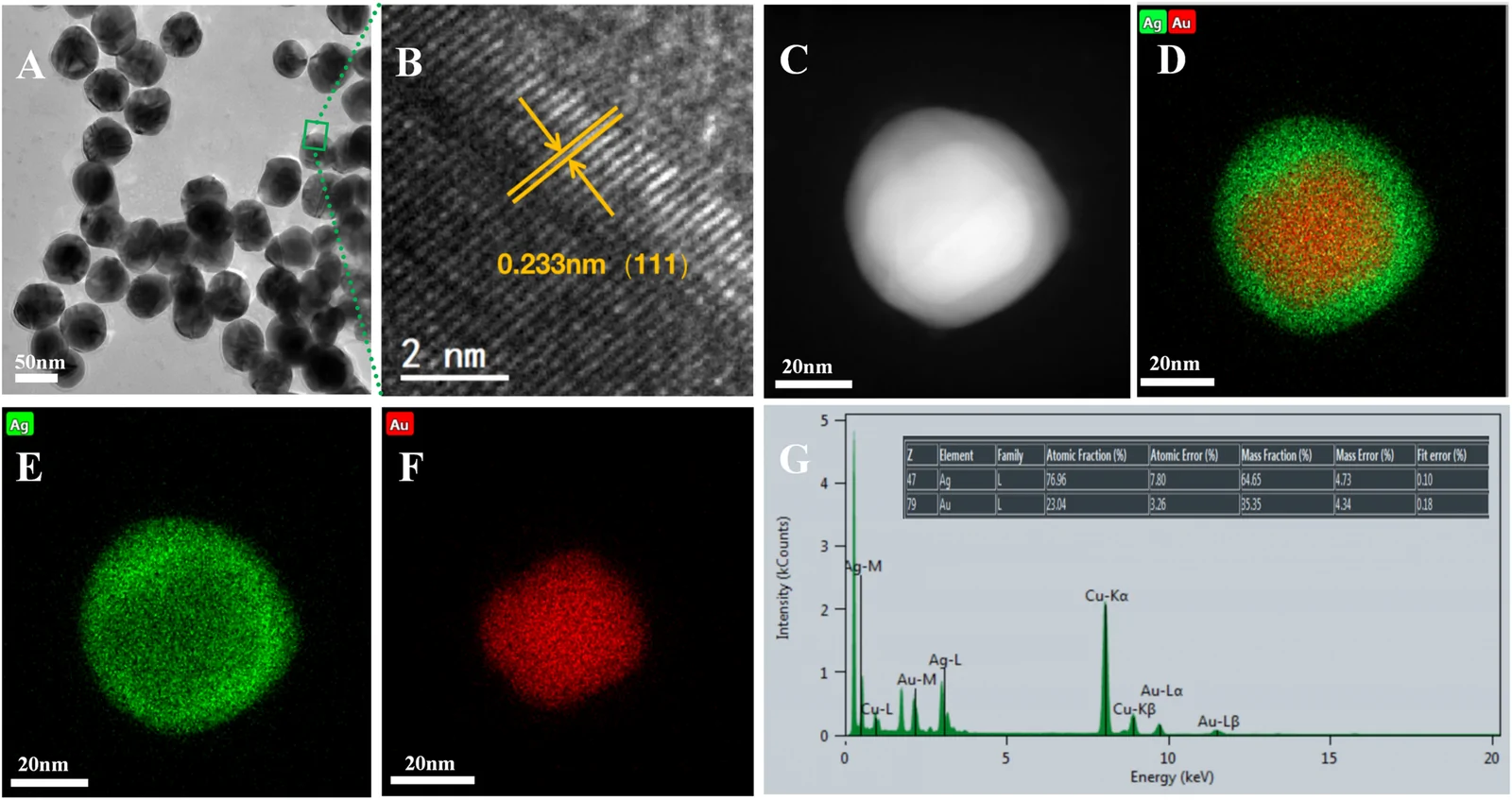
(A) TEM image of the Au@Ag NPs; (B) HRTEM image of the Ag shell region; (C) HAADF-STEM image of the Au@Ag NP; (D)Overlay elemental mapping of Au and Ag; (E) elemental mapping of Ag; (F) elemental mapping of Au; (G) EDS spectrum of Au@Ag NPs.

To directly confirm the successful synthesis of Au@Ag NPs, transmission electron microscopy (TEM) characterization was performed. Fig. 3(A) shows spherical Au@Ag NPs with uniform shape and good dispersion. A high-resolution TEM (HRTEM) image acquired from the Ag-shell region of an individual nanoparticle is presented in Fig. 3(B). An interplanar spacing of 0.233 nm was measured, consistent with the lattice spacing of the (111) plane for fcc Ag (0.2358 nm),^39^ confirming the composition of the shell. This indicates that Ag nanocrystals primarily grew along the (111) orientation on the Au core surface. To further verify the core–shell architecture and elemental distribution at the microscopic scale, high-angle annular dark-field scanning transmission electron microscopy (HAADF-STEM) and associated energy-dispersive X-ray spectroscopy (EDS) analyses were employed. Fig. 3(C) presents a HAADF-STEM image clearly revealing the core–shell structure, characterized by a high-contrast core enveloped by a distinct lower-contrast shell. This significant contrast difference arises from the large atomic-number difference between gold (Au, *Z* = 79) and silver (Ag, *Z* = 47);^40^ the higher atomic number of the Au core generates stronger scattering signals, confirming the core consists of Au. Elemental mapping was conducted to elucidate the spatial distribution of the constituent elements. The presence of both Au and Ag was confirmed by their characteristic peaks in the EDS spectrum (Fig. 3(D)). Corresponding elemental maps (Fig. 3(E and F)) reveal clear spatial distributions: Au (red) is predominantly localized within the core, whereas Ag (green) uniformly coats the core, forming a continuous shell. The consistency between HAADF-STEM image contrast and EDS elemental mapping, combined with simultaneous detection of Au and Ag signals in the EDS spectrum (Fig. 3(G)), directly confirms the successful synthesis of gold-core silver-shell (Au@Ag) nanoparticle structures.

### 3.4 SERS performance

**Fig. 4:**
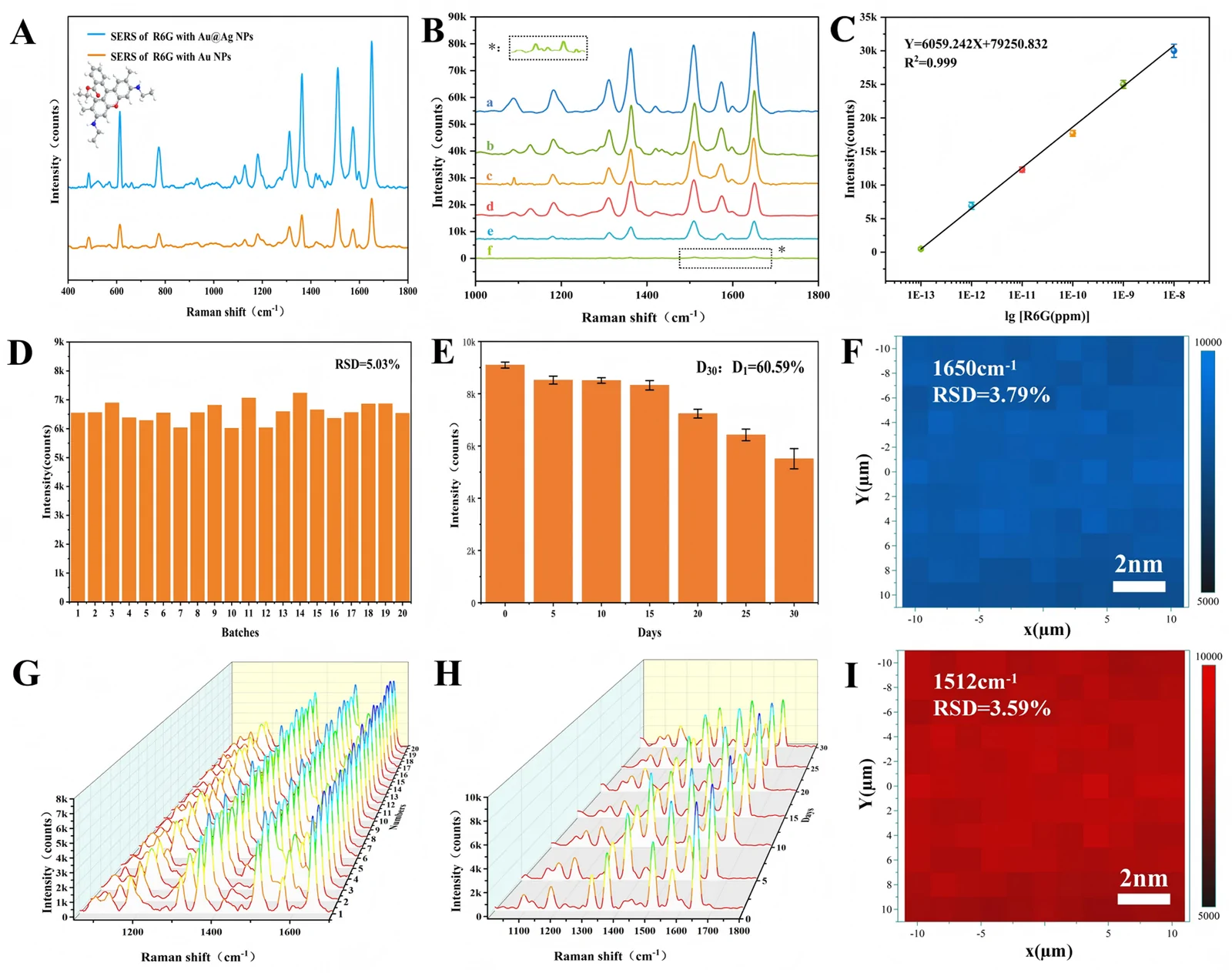
(A) Comparison of SERS performance between Au NPs and Au@Ag NPs; (B) SERS spectra of R6G with different concentrations; (C) linear correlation between the 1650 cm^−1^ intensity and the logarithm of R6G concentration; (D and G) reproducibility of spectral intensity across multiple substrate batches; (E and H) long-term stability test of the same substrates batch; (F and I) Raman mapping images of R6G at 1650 cm^−1^ and 1512 cm^−1^.

The performance evaluation of a SERS substrate typically involves a comprehensive assessment of sensitivity, EF, and uniformity. R6G was selected as a probe molecule due to its characteristically large Raman scattering cross-section and sharp, distinctive peaks. As shown in Fig. 4(A), the blue and orange curves represent the SERS spectra of R6G (10^−8^ M) obtained using Au@Ag NPs and Au NPs, respectively. Evidently, Au@Ag NPs exhibited significantly enhanced Raman signals at all characteristic peaks (*e.g.*, 1362 cm^−1^, 1512 cm^−1^, and 1650 cm^−1^) compared with Au NPs. This substantial signal enhancement is primarily attributed to the synergistic benefits of the core–shell architecture. Firstly, the silver shell provides strong electromagnetic field enhancement due to its superior plasmonic activity in the visible region compared with Au. Secondly, the Au core provides a chemically stable and reproducible seed template for controlled Ag-shell growth, while the outer Ag layer contributes strong plasmonic activity and enhanced electromagnetic fields. Although the exposed Ag shell remains susceptible to oxidation, the Au@Ag configuration provides a practical balance between structural controllability and SERS enhancement for the present application. Furthermore, plasmon coupling at the Au–Ag interface creates additional electromagnetic “hot spots”, amplifying the local field. This design integrates high electromagnetic enhancement with robust chemical stability, explaining the substrate's superior and balanced SERS performance. Consequently, Au@Ag NPs were utilized in all subsequent experiments for further evaluation of their SERS activity.

To directly evaluate the effect of silver shell thickness on SERS activity, the enhancement performance of Au@Ag NPs with varying shell thicknesses was examined using R6G (10^−8^ M). The results, presented in Fig. S1, indicated that SERS signal intensity did not monotonically increase with silver shell thickness but exhibited an optimal value. Au@Ag NPs prepared with 180 µL each of AgNO_3_ and ascorbic acid demonstrated the strongest SERS signals, corresponding to a moderate shell thickness. This thickness achieves an optimal balance between high electromagnetic enhancement from the silver shell and stability from the gold core, likely facilitating strong local electromagnetic “hot spots”. An excessively thin shell may yield insufficient electromagnetic enhancement, whereas an overly thick shell could compromise performance and stability due to silver oxidation or morphological changes.^41^ Consequently, all subsequent experiments in this study employed Au@Ag NPs with this optimized shell thickness as the SERS substrate.

The practical applicability of a SERS substrate significantly depends on its sensitivity, a crucial performance parameter.^42^ In this study, sensitivity was investigated by measuring SERS signals from R6G concentrations ranging from 10^−8^ M to 10^−13^ M. As shown in Fig. 4(B), distinct SERS signals remained detectable even at the lowest tested concentration, demonstrating the high sensitivity of Au@Ag NPs under the present experimental conditions. Within the spectral range of 1000–1800 cm^−1^, several characteristic peaks of R6G were identified: the peak at 1181 cm^−1^ corresponds to in-plane C–H bending vibration, 1311 cm^−1^ to C=C stretching vibration, and 1362 cm^−1^, 1512 cm^−1^, and 1650 cm^−1^ to carbon skeletal stretching modes.^43^ As R6G concentration decreased, SERS signal intensity diminished correspondingly. The characteristic peak at 1650 cm^−1^ was selected to investigate the relationship between SERS intensity and concentration. A robust linear correlation was observed (Fig. 4(C)), described by the regression equation *Y* = 6059.242*X* + 79 250.832 (*R*^2^ = 0.999). The limit of detection (LOD)^33^ was determined based on a signal-to-noise ratio of 3, with the background noise defined as the average intensity at 1621 cm^−1^. The calculated LOD was 8.414 × 10^−14^ M, indicating the high sensitivity of the Au@Ag NP substrate under the present measurement conditions.

The practical utility of SERS technology significantly depends on the stability and reproducibility of the analytical signals. To evaluate the reproducibility of the Au@Ag NPs, multiple independently prepared substrate batches were tested. The calculated relative standard deviation (RSD) of the SERS signal at 1650 cm^−1^ across 20 substrate batches was as low as 5.03% (Fig. 4(D)), indicating excellent batch-to-batch reproducibility. Corresponding SERS spectra (Fig. 4(G)) exhibit consistent peak positions and intensities, confirming the high reproducibility of the substrate fabrication process.

To assess long-term stability, a substrate batch was monitored over one month, with SERS measurements collected at five-day intervals (Fig. 4(E)), and corresponding spectra were recorded (Fig. 4(H)). Although a gradual decrease in signal intensity was observed over the testing period, the substrate demonstrated acceptable long-term stability, retaining 60.59% of its initial signal intensity at 1650 cm^−1^ after 30 days. Additionally, SERS mapping conducted over a 20 × 20 µm^2^ area at characteristic peaks of 1650 cm^−1^ and 1512 cm^−1^ produced uniform intensity distributions (Fig. 4(F) and 4(I)), with exceptionally low RSD values of 3.79% and 3.59%, respectively. This indicates outstanding signal homogeneity across the substrate surface. In addition to homogeneity, the EF^24^ represents another critical performance metric, which was calculated from the peak intensity at 1650 cm^−1^ using the following equation:

1 EF = *I*_SERS_*C*_RS_/*I*_RS_*C*_SERS_

Using *C*_SERS_ = 1.0 × 10^−12^ M and *C*_RS_ = 1.0 × 10^−4^ M, the reference Raman signal was acquired from an R6G droplet deposited on a polished non-SERS-active metal surface. The SERS and reference Raman measurements were performed using the same Raman acquisition conditions described in Section 2.2. Under the assumption of identical sampling geometry and equal sample volumes, the ratio of probed molecule numbers was approximated by the corresponding concentration ratio. Based on this calculation, an estimated EF of approximately 2.78 × 10^8^ was obtained.

### 3.5 SERS analysis of fungicide standard solution

**Fig. 5:**
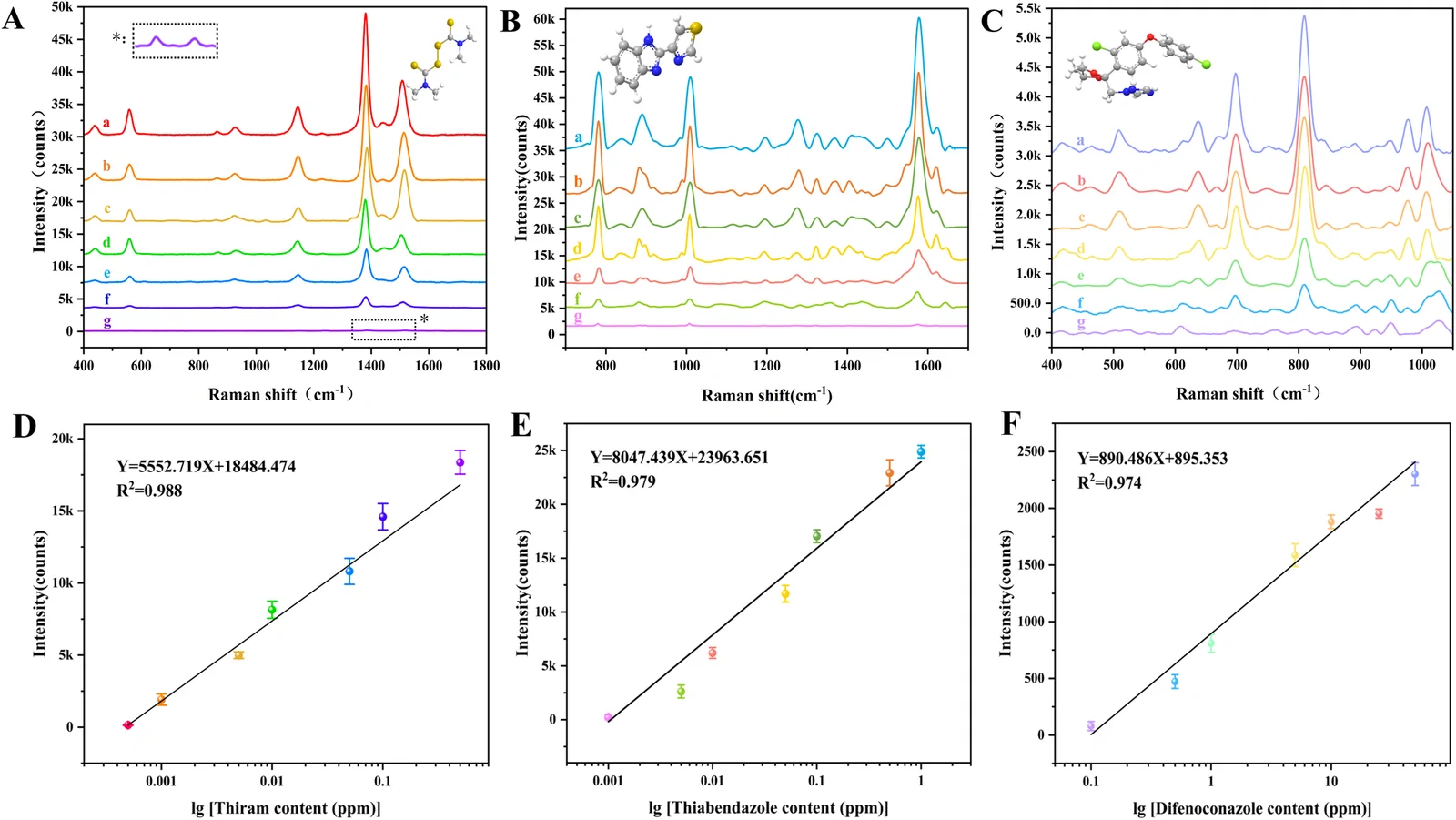
(A) SERS sensitivity detection of thiram; (B) SERS sensitivity detection of thiabendazole; (C) SERS sensitivity detection of difenoconazole; (D) linear relationship between the intensity at 1384 cm^−1^ and the logarithm of thiram concentration; (E) linear relationship between the intensity at 1577 cm^−1^ and the logarithm of thiabendazole concentration; (F) linear relationship between the intensity at 808 cm^−1^ and the logarithm of difenoconazole concentration.

To evaluate the practical applicability of Au@Ag NPs and systematically assess their detection capabilities for individual fungicides in standard solutions, SERS responses for thiram, thiabendazole, and difenoconazole were investigated at varying concentrations.^26^ Using Au@Ag NPs as the SERS substrate, thiram standard solutions ranging from 0.001 to 1 ppm were analyzed (Fig. 5(A)). The spectra clearly show decreasing intensities of characteristic thiram peaks at lower concentrations, yet most peaks remained distinguishable even at 0.001 ppm. The main characteristic peaks were assigned as follows: 562 cm^−1^ corresponds to S–S stretching vibration; 929 cm^−1^ to combined C=S and C–N stretching vibrations; 1146 cm^−1^ and 1514 cm^−1^ originate from C–N stretching and CH_3_ rocking modes, respectively; and the most intense peak at 1384 cm^−1^ arises from C–N stretching coupled with symmetric CH_3_ deformation vibration.^44^ For quantitative analysis of thiram, a calibration curve was established by plotting the intensity at 1384 cm^−1^ against the logarithmic concentration of thiram. A linear relationship described by the equation *Y* = 5552.719*X* + 18 484.474 (*R*^2^ = 0.988) was obtained. Based on this calibration, the calculated LOD for thiram was 0.0047 ppm, highlighting the substrate's promising detection capability. The achieved sensitivity complies with the national regulatory limit (5 ppm),^45^ confirming that the Au@Ag NP-based SERS method is suitable for rapid and sensitive detection of trace-level thiram residues.

Fig. 5(B) displays the SERS detection of thiabendazole standard solutions at various concentrations (0.005–5 ppm). As the concentration decreases, the intensities of the characteristic thiabendazole peaks correspondingly decline; nevertheless, most Raman features remain detectable even at the lowest concentration tested. The peak at 1577 cm^−1^, which shows prominent enhancement in the SERS spectra of thiabendazole,^46^ was selected for quantitative analysis. A calibration curve was established between the peak intensity at 1577 cm^−1^ and the logarithm of thiabendazole concentration. A linear relationship was obtained over the range of 0.005–5 ppm, described by the equation *Y* = 8047.439*X* + 23 963.691 (*R*^2^ = 0.979). Based on this calibration, the corresponding LOD was calculated as 0.0011 ppm, which is significantly below the national regulatory limit of 5 ppm. These results demonstrate that the SERS method based on Au@Ag NPs is suitable for the quantitative analysis of trace-level thiabendazole.

Fig. 5(C) illustrates the SERS spectra of difenoconazole standard solutions at various concentrations obtained using the Au@Ag NP substrate. The SERS spectra of difenoconazole in the concentration range of 0.1–50 ppm are presented. It is clearly observed that the intensity of the characteristic peaks decreases with decreasing concentration. Even at a low concentration of 0.1 ppm, most characteristic Raman signals remain discernible, indicating good detection sensitivity. Two prominently enhanced peaks at 698 cm^−1^ and 808 cm^−1^ in the difenoconazole SERS spectrum were selected for quantitative analysis.^25^ Calibration curves were constructed by plotting the SERS intensity at 808 cm^−1^ against the logarithm of difenoconazole concentration. Within the range of 0.2–10 ppm, a linear relationship was observed, described by the equation *Y* = 890.486*X* + 895.353 (*R*^2^ = 0.974). Based on this calibration, the LOD was calculated as 0.105 ppm, which is lower than the national regulatory limit of 0.2 ppm. These results confirm that the Au@Ag NP-based SERS method provides a reliable platform for sensitive and quantitative detection of trace difenoconazole.

### 3.6 SERS analysis of fungicides on mango surfaces

**Fig. 6:**
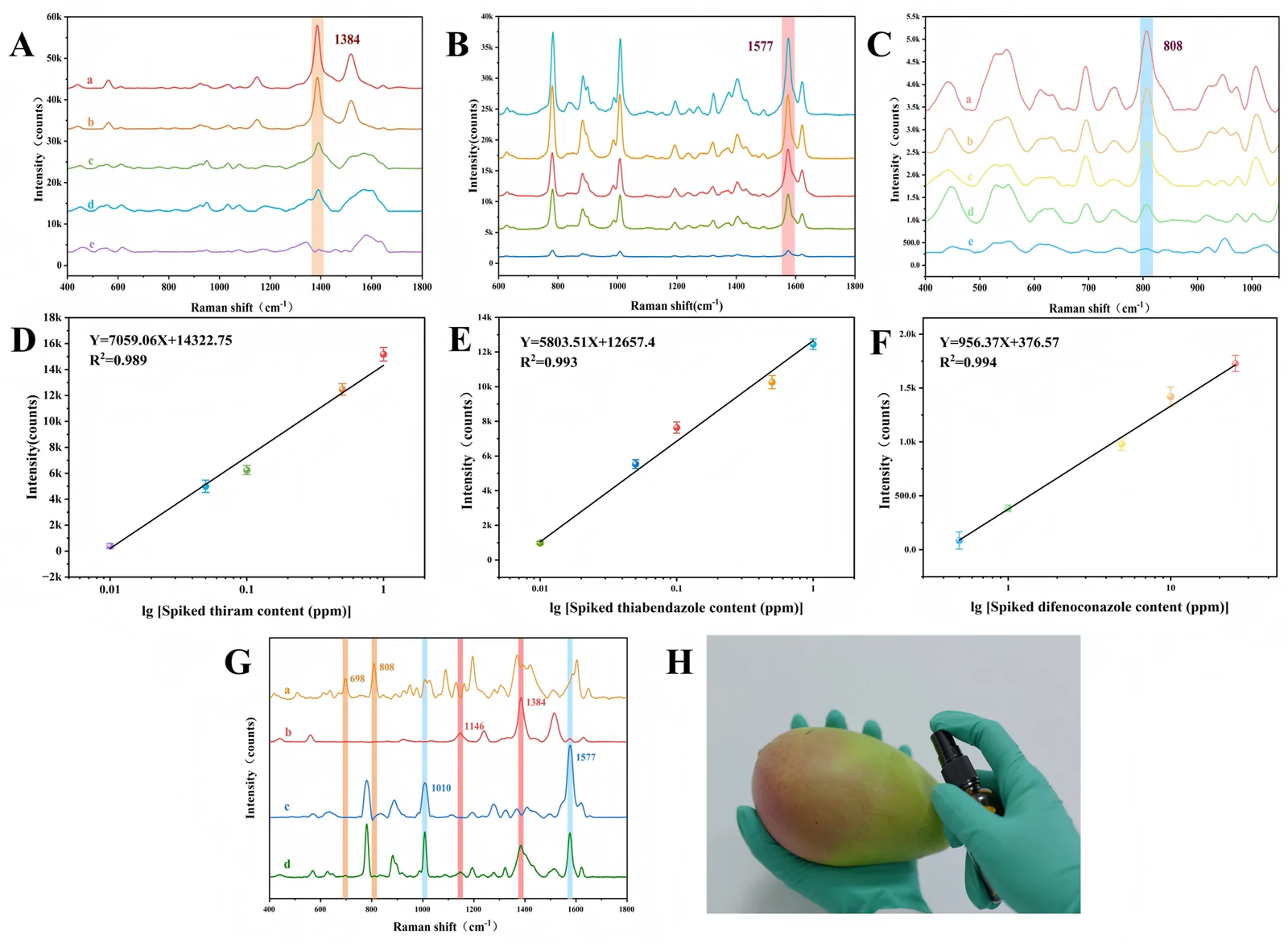
(A) SERS detection of thiram on mango surface; (B) SERS detection of thiabendazole on mango surface; (C) SERS detection of difenoconazole on mango surface; (D) linear relationship between the intensity at 1384 cm^−1^ and the logarithm of spiked thiram concentration; (E) linear relationship between the intensity at 1577 cm^−1^ and the logarithm of spiked thiabendazole concentration; (F) linear relationship between the intensity at 808 cm^−1^ and the logarithm of spiked difenoconazole concentration; (G) SERS detection of three mixed fungicides on mango surface; (H) schematic diagram of SERS detection on mango surface.

In practical applications, SERS substrates must maintain performance within complex matrices. To evaluate substrate effectiveness under realistic conditions, thiram, thiabendazole, and difenoconazole were individually sprayed onto mango surfaces and subsequently analyzed. As shown in Fig. 6(A), within a concentration range of 5–0.01 ppm for thiram, the intensities of its characteristic peaks at 562, 929, 1146, 1384, and 1514 cm^−1^ significantly decreased with reduced concentration, indicating a positive correlation between signal intensity and fungicide concentration. Similarly, as depicted in Fig. 6(B), the intensities of thiabendazole's characteristic peaks at 782, 1010, 1280, and 1577 cm^−1^ diminished progressively as concentrations decreased within the range of 1–0.01 ppm. Likewise, for difenoconazole (25–0.5 ppm), characteristic peaks around 698 and 808 cm^−1^ also displayed a clear concentration-dependent reduction in intensity (Fig. 6(C).

For quantitative analysis of the three fungicides on mango surfaces, calibration curves were established by plotting the characteristic SERS peak intensity against the logarithmic concentration of the corresponding fungicide. For thiram, the characteristic peak at 1384 cm^−1^ was selected for quantitative analysis. As shown in Fig. 6(D), a good linear relationship was obtained, described by the equation *Y* = 7059.06*X* + 14 322.75 (*R*^2^ = 0.989). Similarly, the characteristic peak at 1577 cm^−1^ was selected for quantitative analysis of thiabendazole. The calibration curve shown in Fig. 6(E) exhibited a linear relationship described by the equation *Y* = 5803.51*X* + 12 657.4 (*R*^2^ = 0.993). For difenoconazole, the SERS intensity at 808 cm^−1^ was used to construct the calibration curve, and a linear relationship described by the equation *Y* = 956.37*X* + 376.57 (*R*^2^ = 0.994) was obtained, as shown in Fig. 6(F). These results demonstrate that the characteristic SERS intensities of the three fungicides exhibit good concentration-dependent linear relationships on spiked mango surfaces, providing a quantitative basis for the subsequent recovery analysis.

To further evaluate the quantitative accuracy of the proposed method in the mango matrix, recovery experiments were performed separately for thiram, thiabendazole, and difenoconazole at three spiking levels. The recoveries ranged from 92.24–108.43% for thiram, 92.80–107.64% for thiabendazole, and 82.47–94.85% for difenoconazole, with corresponding RSD values of 4.88–7.58%, 2.84–7.14%, and 5.50–6.98%, respectively (Table 1). These results indicate acceptable accuracy and precision for the individual quantitative determination of the three fungicides in spiked mango surfaces. It should be noted that the recovery experiments were conducted separately for each fungicide and therefore do not represent quantitative validation of the ternary mixture.

**Table 1:** Recovery and precision of thiram, thiabendazole, and difenoconazole in individually spiked mango surfaces

Analyte	Spiked concentration(ppm)	Detected concentration (ppm)	Recovery (%)	RSD (%)
Thiram	1	0.977	97.65	5.77
		1.084	108.43	
		1.078	107.76	
	0.5	0.461	92.24	7.58
		0.531	106.19	
		0.474	94.89	
	0.1	0.103	102.65	4.88
		0.096	95.98	
		0.093	93.45	
Thiabendazole	1	0.935	93.46	7.14
		1.030	103.04	
		1.076	107.64	
	0.5	0.464	92.80	4.40
		0.497	99.31	
		0.505	100.90	
	0.1	0.100	99.96	2.84
		0.096	95.77	
		0.095	94.79	
Difenoconazole	10	8.635	86.35	5.50
		9.485	94.85	
		8.635	86.35	
	5	4.742	94.83	6.98
		4.124	82.47	
		4.486	89.72	
	1	0.943	94.26	6.79
		0.864	86.43	
		0.826	82.57	

The practical applicability of a SERS substrate critically depends on its robustness against complex biological matrices,^47^ which often contain interfering substances like sugars, proteins, and pigments that can suppress Raman signals or generate background noise. Compared to standard solutions, SERS detection on real mango surfaces poses two major challenges. The heterogeneous and rough surface of the mango peel leads to an uneven distribution of dried analytes, while extracted matrix components from the peel can compete for adsorption onto the nanoparticle surface or block SERS hotspots. To evaluate the substrate's performance under these realistic conditions, we first individually sprayed three fungicides onto mango surfaces and analyzed them using the established workflow.^23,47^

Furthermore, the mechanistic distinction between the two strategies explains why sensitivities in real samples are generally lower than those in standard solutions. Kim *et al.* demonstrated that pre-reacting target molecules with nanoparticles (as in our solution-based experiments) significantly enhances SERS performance by maximizing hotspot utilization, whereas sequential deposition (as in mango surface experiments) reduces the probability of analytes occupying hotspots.^48^ In our case, the extraction-assisted approach on mango surfaces relies on re-dissolving dried residues and limited contact between nanoparticles and analytes, which inherently lowers detection efficiency compared to pre-adsorption in solution. This trade-off prioritizes practicality for on-site detection but limits direct quantitative comparison between the two scenarios.

SERS spectra possess fingerprint-like specificity, inherently suitable for multicomponent analysis. Having established the SERS activity of Au@Ag NPs toward the individual fungicides, we further evaluated the feasibility of qualitative multicomponent identification by analyzing a ternary mixture of the three fungicides on mango surfaces. As illustrated in Fig. 6(G), the SERS spectrum acquired from mango surfaces treated with the fungicide mixture clearly exhibited characteristic peaks of all three compounds: thiram (562, 929, 1146, 1384, and 1514 cm^−1^), thiabendazole (782, 1010, 1280, and 1577 cm^−1^), and difenoconazole (700 and 808 cm^−1^). The simultaneous observation of characteristic peaks from all three fungicides demonstrates the feasibility of qualitative multicomponent identification using the Au@Ag NP substrate. Regarding potential spectral overlap, the primary fingerprint peaks for thiram (562, 929, 1146, 1384, and 1514 cm^−1^), thiabendazole (782, 1010, 1280, and 1577 cm^−1^), and difenoconazole (698 and 808 cm^−1^) are well-separated within the spectral window of 500–1650 cm^−1^, with no significant direct overlap. This distinct fingerprint separation represents a key advantage of SERS for multicomponent analyses. Minor proximity of certain peaks (*e.g.*, thiram's 1514 cm^−1^ and thiabendazole's 1577 cm^−1^) did not hinder identification due to differences in relative intensities and the presence of other unique peaks for each fungicide, enabling unambiguous peak assignment. Therefore, the distinct Raman fingerprints of the three fungicides allowed their characteristic peaks to remain distinguishable in the ternary mixture, supporting simultaneous qualitative identification under the tested conditions.

The spraying procedure employed for fungicide application on mango surfaces is illustrated schematically in Fig. 6(H). These results demonstrate the feasibility of simultaneous qualitative identification of thiram, thiabendazole, and difenoconazole in a ternary mixture on mango surfaces. This fingerprint-based identification provides a useful basis for the further development of rapid multi-residue screening methods for agricultural-product surfaces.

To more rigorously position the present method relative to previously reported studies, Table 2 provides a detailed comparison with representative Au@Ag and other related SERS platforms for pesticide analysis. In addition to the detection limit, the comparison includes substrate architecture, target analyte(s), sample matrix, detection mode and sample handling, recovery, reproducibility, and practical format. Previous studies have demonstrated Au@Ag shell-thickness optimization, pesticide detection using Au@Ag substrates in fruit matrices, and SERS-based analysis of multiple pesticide residues. In comparison, the present method combines individual quantitative analysis of thiram, thiabendazole, and difenoconazole with direct application of colloidal Au@Ag nanoparticles to mango surfaces. In addition, the ternary-mixture experiment demonstrates the feasibility of simultaneous qualitative fingerprint identification of the three fungicides. The proposed method therefore offers the combined advantages of simplified sample handling, direct analysis on intact mango peel, sensitive individual quantification of three fungicides, and simultaneous qualitative identification of their ternary mixture, providing a practical and efficient SERS strategy for rapid multi-residue screening on agricultural-product surfaces.

**Table 2:** Comparison of the present Au@Ag NP-based SERS method with representative SERS methods for pesticide analysis in food matrices

Substrate/architecture	Analyte(s)	Sample matrix	Detection performance (LOD)	Recovery/reproducibility	Detection mode/sample handling	References
Au@Ag NP aggregates (tunable Ag shell)	Thiram	Standard solution	1.09 × 10^−9^ M	NR; NR	Colloidal SERS; shell-thickness optimization	37
Au@Ag NP aggregates	Difenoconazole	Grapes	2.8 × 10^−8^ M	NR; NR	QuEChERS-assisted colloidal SERS	41
Au@Ag core–shell NPs (26 nm Au/6 nm Ag)	Thiacloprid, profenofos, oxamyl	Peach	0.1, 0.01, 0.01 mg kg^−1^	78.6–162.0%; NR	Extract-based colloidal SERS	49
2D Au@Ag nanodot array	Thiram, thiabendazole	Fruit juices	0.0011, 0.051 ppm	76–134%; 10.51%	Solid-substrate SERS	50
Au@Ag NPs (this work)	Thiram, thiabendazole, difenoconazole	Mango surfaces	0.0047, 0.0011, 0.105 ppm	THR 92.24–108.43%, TBZ 92.80–107.64%, DIF 82.47–94.85%; 5.03%	Direct surface SERS; ternary qualitative identification	This work

## 4 Conclusion

In this work, core–shell Au@Ag NPs were successfully synthesized and characterized. Their structural features, optical properties, and SERS performance were systematically investigated. The substrate exhibited remarkable SERS performance, demonstrating high sensitivity and a strong linear response toward three widely used fungicides, thiram, thiabendazole, and difenoconazole. The detection limits for these compounds were all below the corresponding national regulatory thresholds. In tests conducted on spiked intact mango surfaces without prior matrix cleanup, the proposed method enabled quantitative analysis of each fungicide individually, indicating that characteristic SERS responses could be obtained despite the presence of native mango-matrix components. The individual recovery experiments yielded recoveries of 92.24–108.43% for thiram, 92.80–107.64% for thiabendazole, and 82.47–94.85% for difenoconazole, with RSD values ranging from 2.84% to 7.58%, supporting the accuracy and precision of the individual quantitative analyses. This demonstrates a certain degree of tolerance to matrix interference under the tested conditions. In addition, the characteristic Raman fingerprints of thiram, thiabendazole, and difenoconazole could be simultaneously identified in a ternary mixture. These results demonstrate the feasibility of combining individual quantitative analysis with qualitative multicomponent identification using the Au@Ag NP-based SERS strategy. The Au@Ag NP-based SERS method provides an efficient and sensitive analytical platform for rapid on-site screening of multiple fungicide residues in agricultural products, highlighting the promising potential of core–shell Au@Ag NPs in food safety monitoring. Future studies may focus on further structural optimization to enhance detection performance and on extending this approach to the detection of other classes of contaminants. Furthermore, to advance this technology toward practical, field-deployable applications, future work will explore the development of solid-state SERS sensors, for example by immobilizing Au@Ag NPs on flexible films or chip-based platforms, and integrating them into portable or handheld Raman devices. These directions are critical for transforming the solution-based sensing strategy presented here into a robust, user-friendly tool for rapid on-site screening in agricultural settings.

## Author contributions

Ziwen Zhang: conceptualization; software; data curation; writing – original draft; methodology. Weiye Yang: supervision; writing – review & editing; methodology. Dongan Wu: writing – original draft, visualization, validation, software, data curation. Ping Liang: formal analysis, data curation. Hongyan Peng: conceptualization, formal analysis, data curation. Lijian Meng: software, formal analysis. Shihua Zhao: conceptualization; supervision; writing – review & editing funding acquisition; investigation; methodology; project administration; resources.

## Conflicts of interest

The authors declare no competing interests.

## Supplementary Material

RA-OLF-D6RA05546J-s001

## Data Availability

The data supporting this study are currently hosted in a private Mendeley Data repository, accessible *via* this preview link for the purpose of editorial and peer review: https://data.mendeley.com/datasets/2nns3vy2b2/1. Upon acceptance of the manuscript, this repository will be made public to all readers. Supplementary information (SI): SERS signal comparison of Au@Ag NPs with different silver shell thicknesses using R6G (Fig. S1). See DOI: https://doi.org/10.1039/d6ra05546j.
