# Supplementary material for: Core–shell Au@Ag nanoparticles as a high-performance SERS substrate for detection of multiple fungicides on mango surfaces
Source: RSC Adv. 2026 Sep 17;16(44):55566–78. doi: 10.1039/d6ra05546j (PMC13583630; doi:10.1039/d6ra05546j)
Supplement: Supplementary file 1 [file RA-016-D6RA05546J-s001.pdf]

Supplementary Information (SI) for RSC Advances.
This journal is © The Royal Society of Chemistry 2026

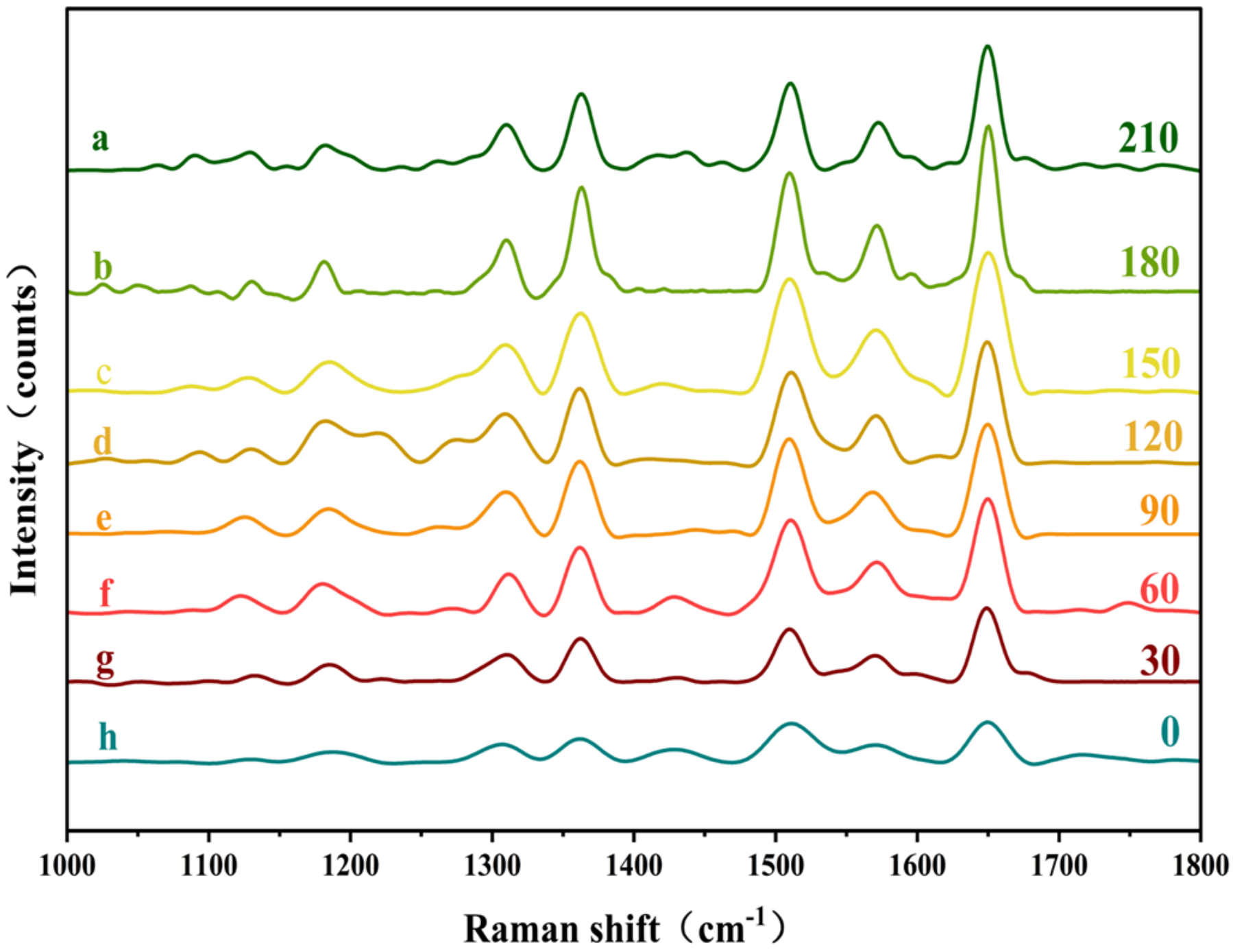

Figure S1 SERS spectra of 10^{-8} M R6G adsorbed on Au@Ag NPs with different Ag shell thicknesses.
